# The effect of implants loaded with stem cells from human exfoliated deciduous teeth on early osseointegration in a canine model

**DOI:** 10.1186/s12903-022-02264-5

**Published:** 2022-06-17

**Authors:** Xu Cao, Caiyun Wang, Dingxiang Yuan, Su Chen, Xin Wang

**Affiliations:** Laboratory of Biomaterials and Biomechanics, Beijing Key Laboratory of Tooth Regeneration and Function Reconstruction, Beijing Stomatological Hospital, Capital Medical University, Beijing, China

**Keywords:** Animal models, Dental implants, Stem cell transplantation, Stem cells from human exfoliated deciduous teeth (SHEDs), Osseointegration

## Abstract

**Background:**

This in vivo experimental study investigated the effect of stem cells from human exfoliated deciduous teeth (SHEDs) on early osteogenesis around implants.

**Methods:**

In four healthy adult male Beagle dogs, the left mandibular received implants and SHED as the experimental group, and the right mandibular received implants and phosphate-buffered saline as the control group. The Beagle dogs were randomly divided into groups A and B, which were sacrificed at 2 and 4 weeks after implantation. Micro-computed tomography and histological analysis were used to investigate the effect of SHED-loading on the early osseointegration around the implants.

**Results:**

The total bone-to-implant contact (BIC%) and interthread bone improved significantly. The analysis of the bone volume fraction and trabecular thickness showed that the bone trabecula around the implants in the SHEDs group was thicker and denser than that in the control group, suggesting a better osseointegration.

**Conclusions:**

The application of implants pre-adhered with SHEDs improved and accelerated early osseointegration around the implant, resulting in thicker and denser trabecular bone.

## Background

With the continuous improvement of stomatology and biomaterials science, oral implants have become the most effective way to replace missing teeth.

Osseointegration, the term for the integration of bone and implant material [1], is the key to the success of implants [2]. However, in some patients, implants fail due to poor early osseointegration, with an incidence of approximately 2% in the first few months after implantation [3, 4], while in others they may fail due to an extended healing time. Thus, methods to accelerate the rate of osseointegration to shorten the healing time after implant placement and improve the long-term stability of the implant is of great importance in current research.

The main factors affecting osseointegration include dental materials, designs, and surface topographies of the dental implants [5]. Previous studies mainly focused on surface modification of the implant, such as chemical etching, to increase the hydrophilicity and surface roughness of the implant, which can promote the proliferation and differentiation of osteoblasts or human periodontal ligament stem cells (hPDLSCs) and accelerate the speed of osseointegration [6–10]. Other surface modifications include hydroxyapatite coatings, such as calcium phosphates, but some studies have shown that calcium phosphate deposited on implants do not improve early tissue integration [11, 12] and may lead to infection and adverse tissue reactions, including rapid negative bone resorption [13, 14].

Several studies have shown that increasing the proliferation, migration, and differentiation of osteoblasts or mesenchymal stem cells (MSCs) can promote osteogenesis [8, 15, 16]. Increasing the number of functional cells in the osseointegration area has been shown to effectively improve osseointegration [17]. De Bruijn et al. [18] demonstrated that titanium implants coated with bone marrow cells and implanted subcutaneously into nude mice could form bone tissue on their surfaces, but they did not investigate whether these implants could accelerate bone formation at the site of tissue formation. Stuermer et al. [16] found that autologous osteoblast coating could accelerate and enhance the osseointegration of titanium implants. However, it is inconvenient to obtain autologous osteoblasts, and there are still some ethical issues; the source of functional cells needs to be improved.

Stem cells from human exfoliated deciduous teeth (SHEDs) are considered a type of MSC because they are derived from the neural crest. SHEDs have multidirectional differentiation and self-renewal functions, and can differentiate into osteoblasts, odontoblasts, chondrocytes, hepatocytes, adipocytes, neuronal cells, and so on [19]. Changing the cell culture microenvironment can regulate their proliferation and differentiation into different types of functional cells [20], and they have immune-phenotypes similar to bone marrow mesenchymal stem cells (BMMSCs) [21]. They can be cryopreserved at a low temperature for an extended period and maintain good cell viability [22–26]. Because of their easy accessibility and astonishing cell numbers, they are becoming an ideal source of MSCs [19]. Compared with other types of MSCs, SHEDs have a higher proliferation rate and enhanced osteoinductive ability in vivo [27–29] and are more convenient to obtain than other MSCs, making them a potentially important cell source in bone regeneration therapy [30].

Therefore, in this study, we utilized implants with pre-adhered SHEDs to evaluate whether SHEDs could decrease the healing time of osseointegration to provide a possible strategy for the long-term stability of the implants.

## Methods

### Study design

This preclinical study trial included two healing periods (2 and 4 weeks after implant placement) to compare the effects of SHEDs on peri-implant osteogenesis in beagle dogs. The research includes the following interventions: (1) preparation of SHEDs, (2) surgical procedures, (3) micro-CT analysis, (4)histological processing, (5) histomorphometric analysis.

### Experimental sample

Four healthy, 1-year-old male Beagle dogs were purchased from Fangyuanyuan Co., Ltd (Beijing, China) and were randomly divided into groups A and B. Group A dogs underwent experimental observation for 2 weeks, while those in group B were observed for 4 weeks. In each Beagle dog, the left mandibular received implants and SHED as the experimental group, and the right mandibular received implants and phosphate-buffered saline (PBS) as the control group. The experimental group and the control group each included 6 implants at each time point.

The Animal Ethical and Welfare Committee of the Beijing Stomatological Hospital (Beijing, China) approved the study protocol (Approval No. KQYY-201909-002). All methods in the study were performed in accordance with the ARRIVE guidelines and the Directive 2010/63/EU in Europe. The animals were housed in the Beijing Stomatological Hospital (Beijing, China), and all surgeries were performed by the same surgeon. All experiments were performed according to the regulations about care and use of research animals. All beagle dogs were adaptively fed for 3 weeks prior to the start of the experiment.

### Study devices

Zimmer Tapered Screw-Vent implants (TSV, Zimmer Biomet, USA) with a diameter of 3.7 mm and a length of 8 mm were used in this study.

### Preparation of SHEDs

**Fig. 1:**
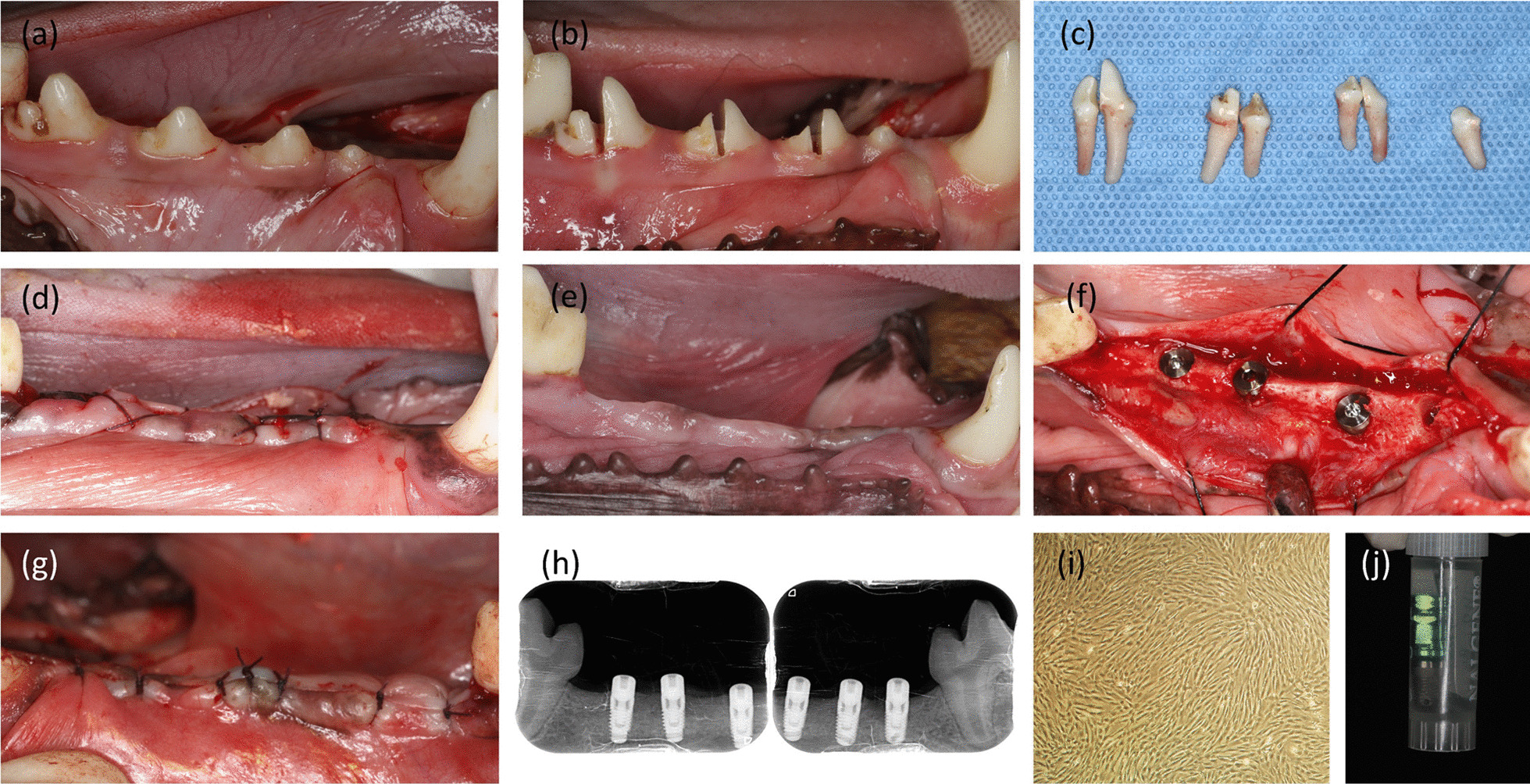
The clinical steps of the experiment. **a** Baseline situation. **b** Teeth hemi-section prior to extraction. **c** Teeth extraction. **d** Suture after teeth extraction. **e** Healed crest three months after extractions. **f** Implant placement. **g** Suture after implant placement. **h** X-ray of mandible. **i** The image of the SHEDs (×40). **j** Placement of the implants in the centrifuge tube containing SHEDs cell suspension prior to implantation

The SHEDs (Kati, shanghai, China) were expanded for passage (Fig. 1), and the medium was aspirated and rinsed thrice with PBS. Following trypsinization, the cells were incubated at 37 °C for 2 min, transferred to a new centrifuge tube, and centrifuged at 1100 rpm for 10 min. After discarding the supernatant, we resuspended the centrifuged SHEDs in PBS at an aliquot size of 1 × 10^6^/50 uL or 2 × 10^6^/1000 uL. The implants in the experimental group were immersed in the cell suspension containing SHEDs at a cell density of 2 × 10^6^/1000 uL.

### Surgical procedures

After general anesthesia with the recommended dose of Sumianxin II (0.08–0.1 mL/kg) combined with half the recommended dose of 3% sodium pentobarbital (0.5 mL/kg) by intramuscular injection, the dogs were placed in the supine position and routinely draped for disinfection. Minimally invasive extraction of P1, P2, P3, and P4 premolars of both mandible sides was performed, and a collagen sponge was placed in the extraction socket. The wound was closed using the interrupted suture technique with 4–0 absorbable sutures (ETHICON, USA). Following the procedure, dogs received intramuscular injections of penicillin for three days and were fed semiliquid food for 1 week.

Three months after the extraction wound healed, implant placement was performed using the same preoperative anesthesia regimen as was used for the tooth extraction procedure. A horizontal incision was made on the top of the alveolar ridge in the missing tooth area. The full-thickness flaps were elevated to expose the bone surface of the tooth to be implanted. The implant socket was fixed, positioned, and gradually prepared under 0.9% saline cooling. After the cavity preparation, 50 uL of SHED cell suspension (cell number 1 × 10^6^) was injected into the cavity of the experimental side, and 50 uL PBS was injected into the cavity of the control side. Three implants (Zimmer Biomet, USA) were implanted in the mandibular premolar area on each side according to the bone mass of the implant area (Fig. 1). The initial stability of the implants was ensured, and the implant torque was 35–50 N·cm. After placing the closing screws, the full-thickness buccal and lingual flaps were repositioned and sutured tightly without tension. The condition of the implant and its surrounding soft tissues were checked daily for seven days after the operation, and the dogs’ mouths were washed with 1:5000 chlorhexidine solution with simultaneous use of a saliva suction device to maintain a clear airway. Following the procedure, dogs received intramuscular injections of penicillin for three days and were fed semiliquid food for 1 week.

The Beagle dogs were sacrificed with overdose pentobarbital sodium (120 mg/kg/i.v.) at two and 4 weeks [31] after implantation. The mandible was sawed from the mandibular angle using a sterile bone saw. The mandibular body was completely removed and immediately placed in a specimen bag containing a 10% neutral formaldehyde solution for later use. Prior to histological processing, the implants were individually separated for micro-computed tomography (CT) scanning.

### Micro-CT analysis

Micro-CT (100 kv, 50 μAz, SkyScan1276, Bruker) [32] was used for imaging examination. A VOI of 3 mm of length and 4.7 mm of diameter was selected from the first screw of the implant. CTAn software was used to analyze the results. The following outcomes were measured: (a) bone volume fraction (percent bone volume, BT/TV); (b) trabecular thickness (TbTh); (c) trabecular number (TbN); and (d) trabecular separation/ spacing (TbSp).

### Histological processing

The samples were dehydrated in a series of ethanol solutions (70%–95%) and embedded in a light-curing resin (Technovit 7, 200 VLC, Japan). The resin blocks containing the implant were sliced using a German EXAKT300CP hard tissue slicer (EXAKT, Germany) in the buccal and lingual direction and then polished using 320, 800, 1200 and 4000 grit silicon carbide paper. The final tissue thickness was approximately 30 μm. Methylene blue acid fuchsin staining was used to observe the osseointegration of the implant.

### Histomorphometric analysis

**Fig. 2:**
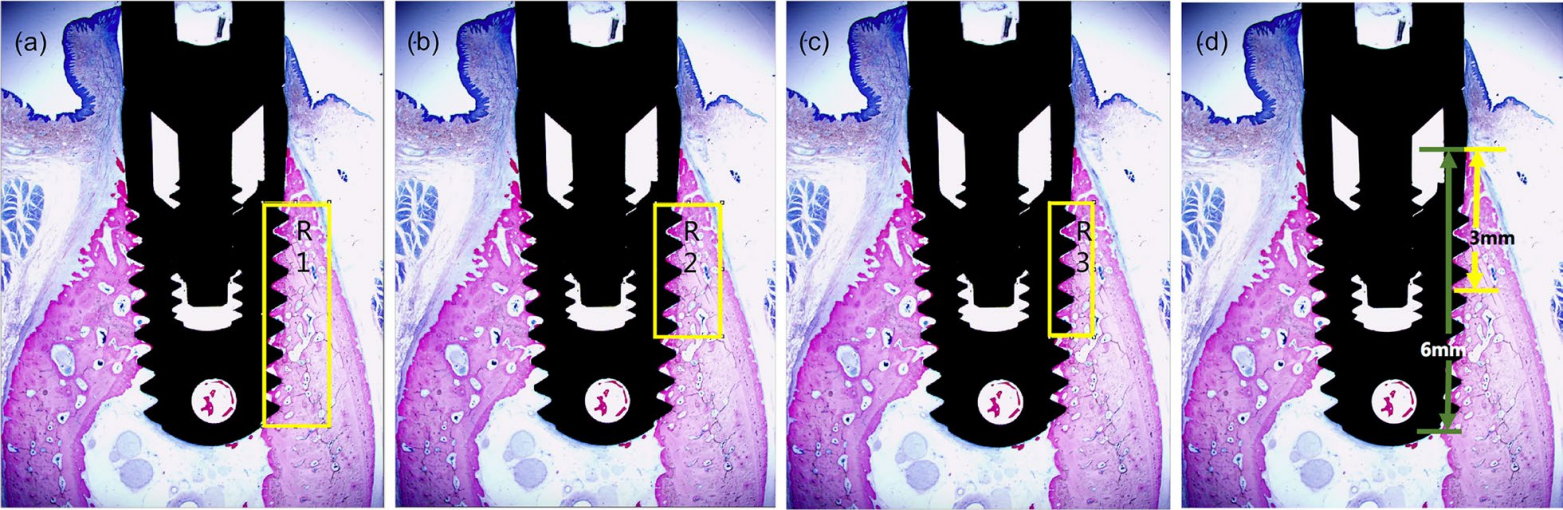
Schematic diagram. **a** R1 represents the bone volume fraction in the area 1.5 mm wide and 5 mm long from the inner thread of the implant. **b** R2 represents the bone volume fraction in the area 1.5 mm wide and 3 mm long from the inner thread of the implant. **c** R3 represents the bone volume fraction in the area 1 mm wide and 3 mm long from the inner thread of the implant. **d** Histological bone-to-implant contact measurement. Coronal bone in contact (BIC): bone in contact with implant surface within 3 mm from the most coronal bone contact. Total BIC: bone in contact with implant surface within 6 mm from the most coronal bone contact

The histometric evaluation was carried out using Image J software. The peri-implant bone fraction (BF), bone-to-implant contact (BIC%), and interthread bone (IB) were assessed [33–38]. The peri-implant BF was defined as the percentage area of bone tissues within a rectangular area region situated from the axis of the implant (Fig. 2a–c). Coronal BIC% [34] was defined by bone in contact with the implant surface within three millimeters of the most coronal bone contact, whereas total BIC% was defined by bone in contact with the implant surface within six millimeters of the most coronal bone contact. (Fig. 2d). Finally, IB [35] was considered as the percentage area of bone tissue between all threads of the implant.

### Statistical analysis

Data from both histological and micro-CT analysis are expressed in means ± standard deviation (*SD*). All statistical were performed with SPSS 26. The statistical significance was determined by the student’s *t*-test, and *p* values < 0.05 were considered as significant.

## Results

### Clinical findings

All dogs in the study experienced an uneventful course of healing, with no loosened or missing implants.

### Histological findings

**Fig. 3:**
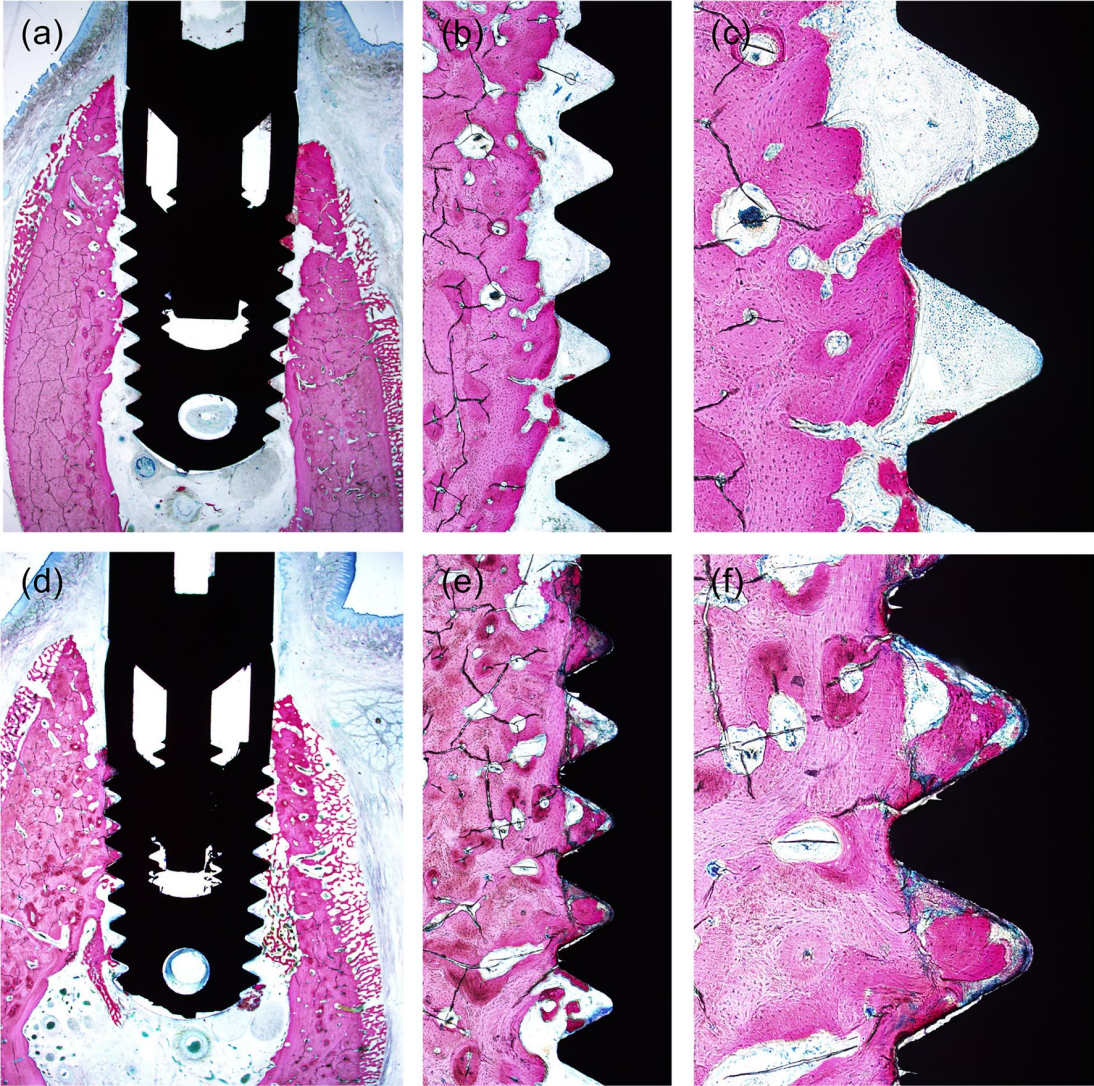
Two weeks post-implantation. Control group **a** ×12.5; **b** ×40; **c** ×100. SHEDs group **d** ×12.5; **e** ×40; **f** ×100. SHEDs, Stem cells from human exfoliated deciduous teeth

The results of healing at 2 weeks post implantation are shown in Fig. 3. A gap in the crestal site between the implant and the bone surface was still apparent. In the SHEDs group, bone remodeling and osteoid deposition were observed on both the bone and implant surfaces, with new cancellous bone formation occurring between the implant and bone surface.

**Fig. 4:**
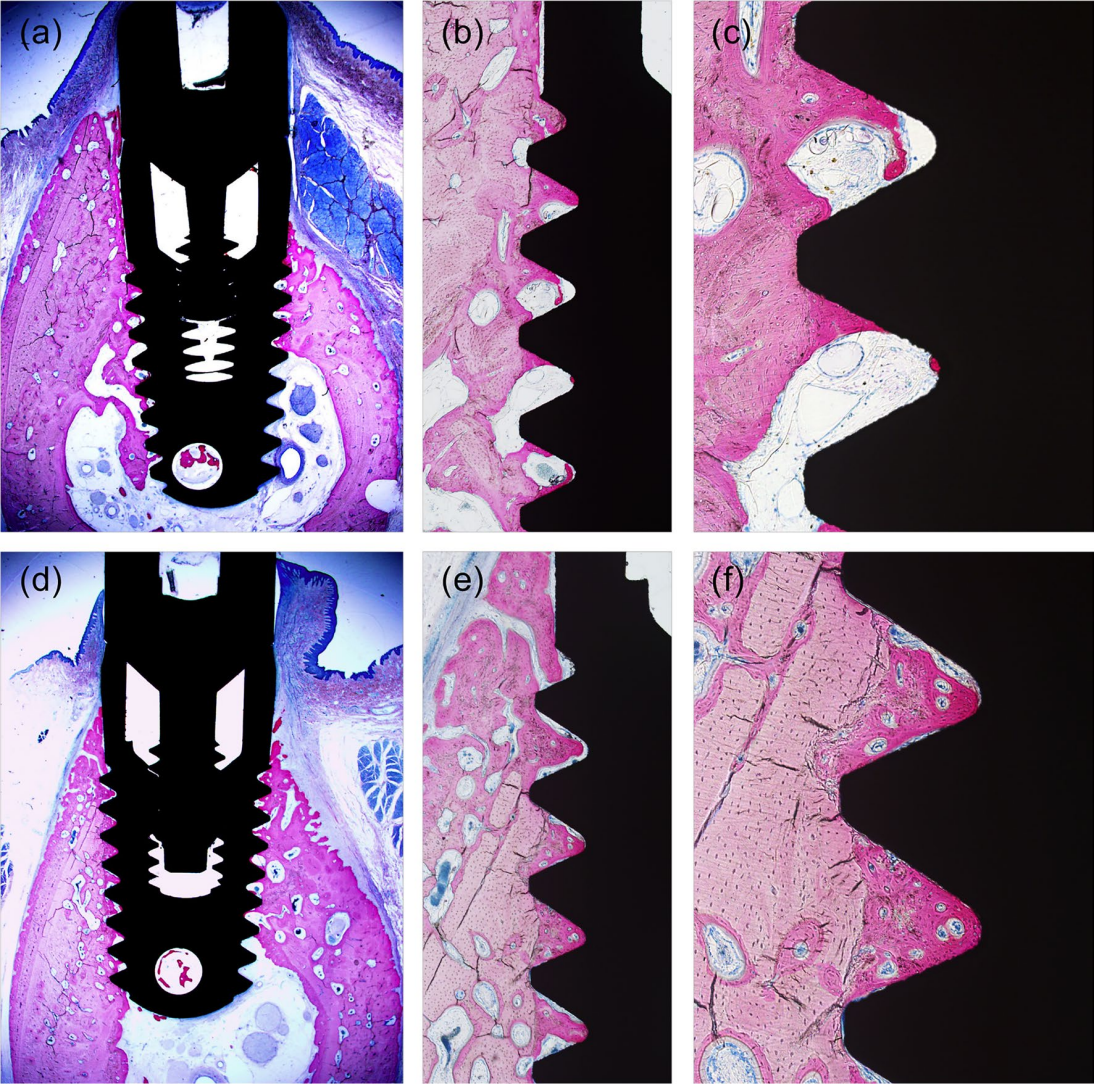
Four weeks post-implantation. Control group **a** ×12.5; **b** ×40; **c** ×100. SHEDs group **d** ×12.5; **e** ×40; **f** ×100. SHEDs, Stem cells from human exfoliated deciduous teeth

The results of healing at 4 weeks post implantation are shown in Fig. 4. In the SHEDs group, there was an absence of intervening fibrous tissue; close contact between the bone and the implant surface was observed (Fig. 4f), and there was more bone in the SHEDs group than in the control group.

### Histometric results

**Fig. 5:**
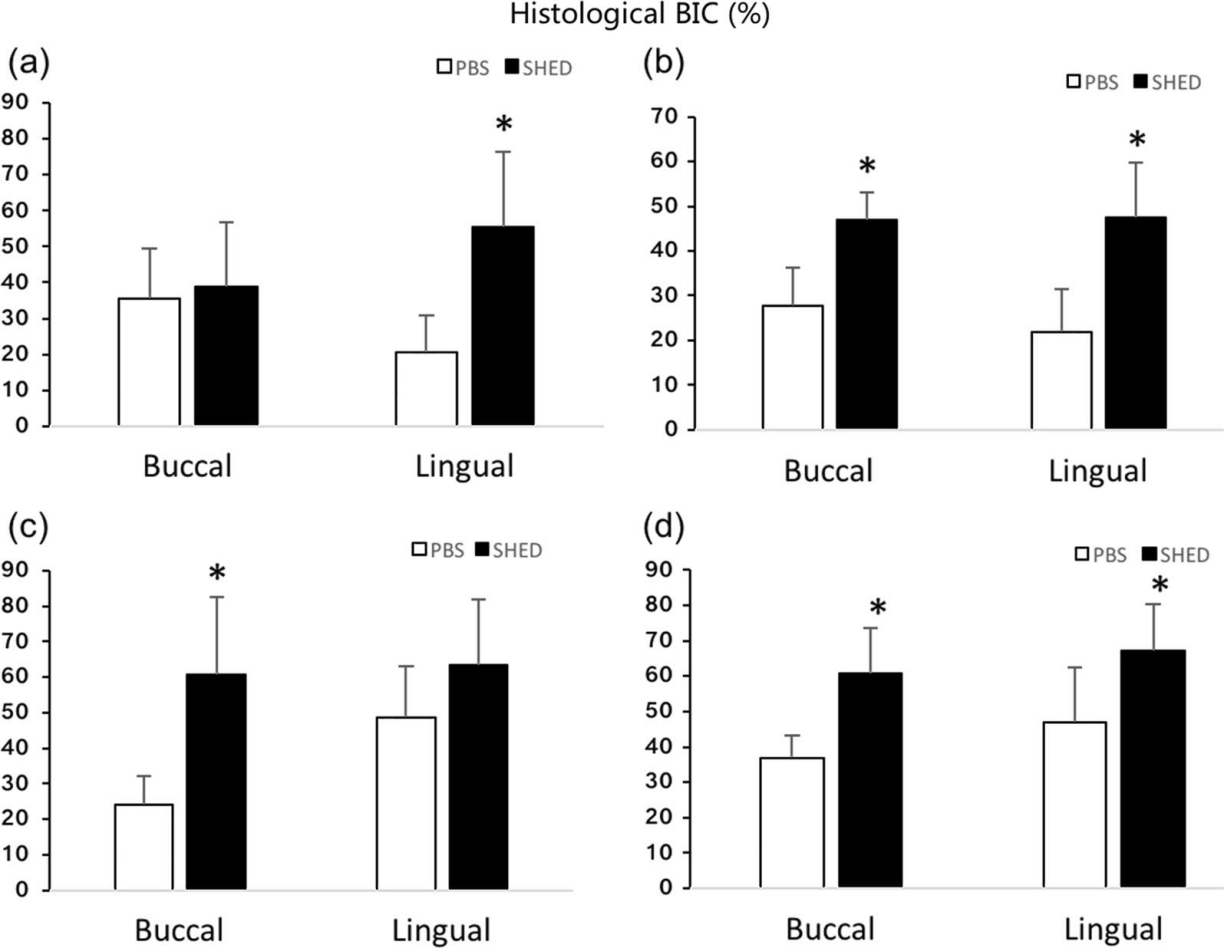
Bone in contact (BIC) results. **a** Coronal BIC at 2 weeks post implantation. **b** Total BIC at 2 weeks post implantation. **c** Coronal BIC at 4 weeks post implantation. **d** Total BIC at 4 weeks post implantation. *p < 0.05

The results for the Coronal BIC% and Total BIC% are shown in Fig. 5 and Table 1. At 2 weeks post-implantation, the Coronal BIC% was increased on the lingual side (55.29 ± 21.22%), and the Total BIC% was increased on both the buccal (46.92 ± 6.22%) and lingual sides (47.38 ± 12.4%) in the SHEDs group.

**Table 1:** Bone in contact (BIC) in different groups: 2-week healing, 4-week healing in buccal and lingual (mean ± *SD*)

Group	Coronal BIC Buccal 2 W (%)	Coronal BIC Lingual 2 W (%)	Total BIC Buccal 2 W (%)	Total BIC Lingual 2 W (%)	Coronal BIC Buccal 4 W (%)	Coronal BIC Lingual 4 W (%)	Total BIC Buccal 4 W (%)	Total BIC Lingual 4 W (%)
PBS	35.47 ± 14.07	20.39 ± 10.61	27.79 ± 8.60	21.92 ± 9.42	24.12 ± 8.19	48.72 ± 14.49	36.82 ± 6.41	46.88 ± 15.44
SHED	38.80 ± 17.87	55.29 ± 21.22	46.92 ± 6.22	47.38 ± 12.40	60.86 ± 21.71	63.47 ± 18.42	60.73 ± 12.75	67.28 ± 13.01

**Fig. 6:**
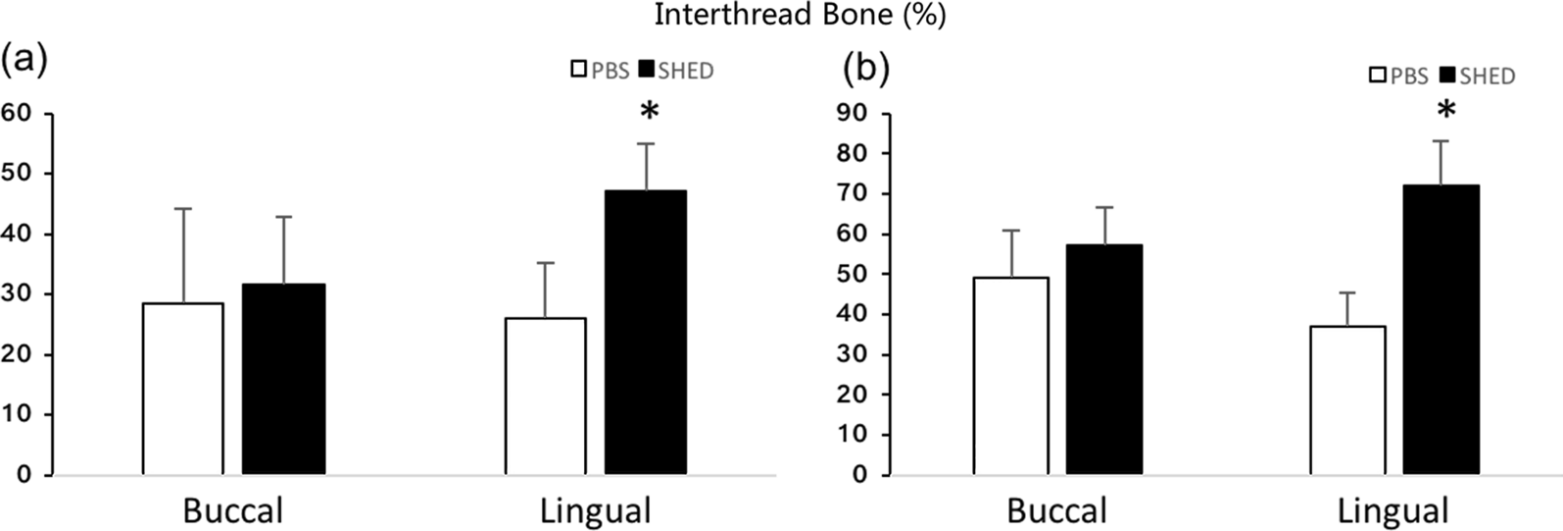
The interthread bone (IB) results. **a** The IB at 2 weeks post implantation. **b** The IB at 4 weeks post implantation. *p < 0.05

The IB results are shown in Fig. 6 and Table 2. At 2 weeks post-implantation, there was significant difference in IB on the lingual side between the SHEDs group (47.23 ± 7.8%) and the control group (26.1 ± 9.2%). At 4 weeks post-implantation, the IB was further increased over that at 2 weeks, and there was a significant difference between the SHEDs group (72.14 ± 11.15%) and the control group (37.12 ± 8.21%) on the lingual side.

**Table 2:** The interthread bone (IB) in different groups: 2-week healing, 4-week healing in buccal and lingual

Group	IB Buccal 2 W (%)	IB Lingual 2 W (%)	IB Buccal 4 W (%)	IB Lingual 4 W (%)
PBS	28.41 ± 15.82	26.10 ± 9.20	49.19 ± 11.79	37.12 ± 8.21
SHED	31.65 ± 11.23	47.23 ± 7.80	57.06 ± 9.46	72.14 ± 11.15

**Fig. 7:**
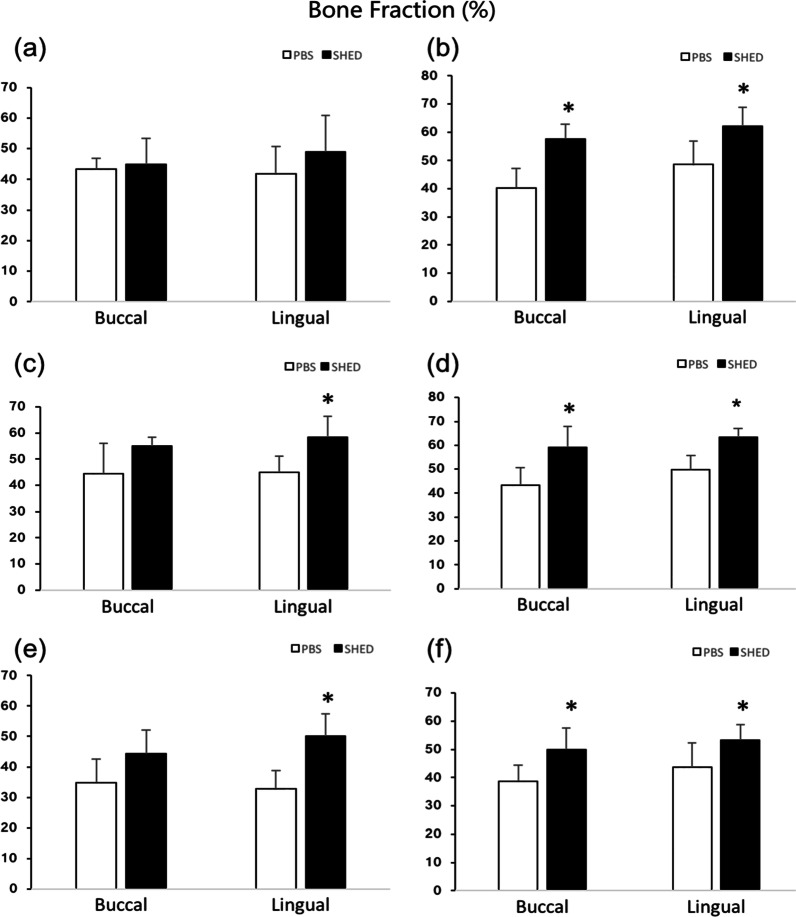
Peri-implant bone fraction (BF) (%). **a** R1 at 2 weeks. **b** R1 at 4 weeks. **c** R2 at 2 weeks. **d** R2 at 4 weeks. **e** R3 at 2 weeks. **f** R3 at 4 weeks. *p < 0.05

The results of peri-implant BF are shown in Fig. 7, Tables 3 and 4. At 2 weeks post-implantation (Table 3), the BF was higher in the SHEDs group in R2 (58.29 ± 8.24% vs. 44.99 ± 6.08%) and R3 (50.08 ± 7.28% vs. 32.87 ± 5.84%) on the lingual side compared with that in the control group, but there was no significant difference on the buccal side, although there was a slight increase in R3 in the SHEDs group.

**Table 3:** Peri-implant bone fraction (BF) (%) in different groups: 2-week healing in buccal and lingual

Group	BF-RI Buccal 2 W (%)	BF-RI Lingual 2 W (%)	BF-R2 Buccal 2 W (%)	BF-R2 Lingual 2 W (%)	BF-R3 Buccal 2 W (%)	BF-R3 Lingual 2 W (%)
PBS	43.38 ± 3.64	41.89 ± 8.85	44.42 ± 11.61	44.99 ± 6.08	34.87 ± 7.71	32.87 ± 5.84
SHED	45.07 ± 8.35	49.03 ± 11.85	54.94 ± 3.41	58.29 ± 8.24	44.34 ± 7.84	50.08 ± 7.28

**Table 4:** Peri-implant bone fraction (BF) (%) in different groups: 4-week healing in buccal and lingual

Group	BF-RI Buccal 4 W (%)	BF-RI Lingual 4 W (%)	BF-R2 Buccal 4 W (%)	BF-R2 Lingual 4 W (%)	BF-R3 Buccal 4 W (%)	BF-R3 Lingual 4 W (%)
PBS	40.21 ± 7.06	48.56 ± 8.26	43.38 ± 7.37	49.82 ± 5.84	38.53 ± 5.88	43.62 ± 8.64
SHED	57.49 ± 5.26	62.17 ± 6.64	59.22 ± 8.58	63.26 ± 3.79	49.95 ± 7.69	53.22 ± 5.50

At 4 weeks post-implantation (Table 4), the BF in R1(57.49 ± 5.26% vs. 40.21 ± 7.06% in buccal side), R2(59.22 ± 8.58% vs. 43.38 ± 7.37% in buccal side), and R3(49.95 ± 7.69% vs. 38.53 ± 5.88% in buccal side) was significantly higher on both the buccal and lingual sides (R1 62.17 ± 6.64% vs. 48.56 ± 8.26%; R2 63.26 ± 3.79% vs. 49.82 ± 5.84%; R3 53.22 ± 5.50% vs. 43.62 ± 8.64%) in the SHEDs group compared with that in the control group. Moreover, the BF on the lingual side was slightly higher than that on the buccal side.

### Micro-CT results

**Fig. 8:**
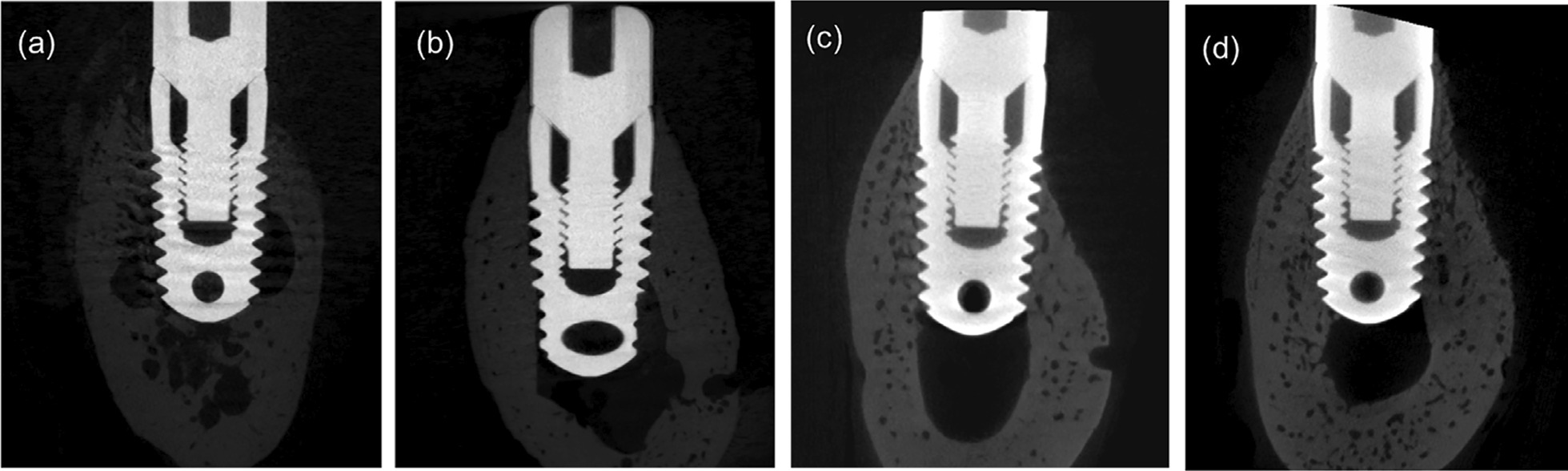
Micro-computed tomography images. **a** Control group at 2 weeks. **b** Experimental group at 2 weeks. **c** Control group at 4 weeks. **d** Experimental group at 4 weeks

**Fig. 9.**
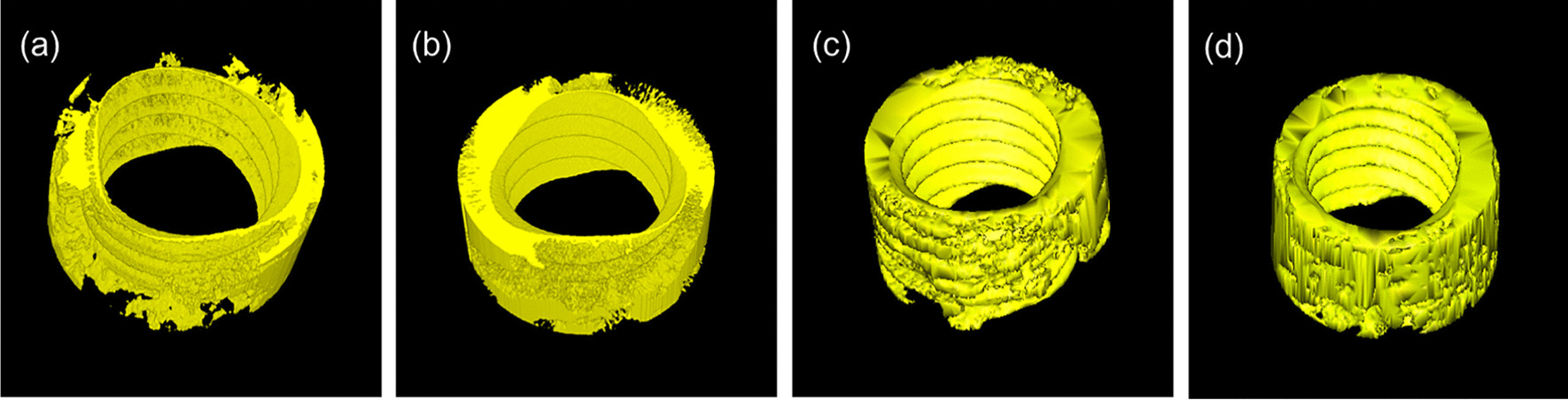
3D reconstruction of the bone around the implant. **a** Control group at 2 weeks. **b** Experimental group at 2 weeks. **c** Control group at 4 weeks. **d** Experimental group at 4 weeks

**Fig. 10:**
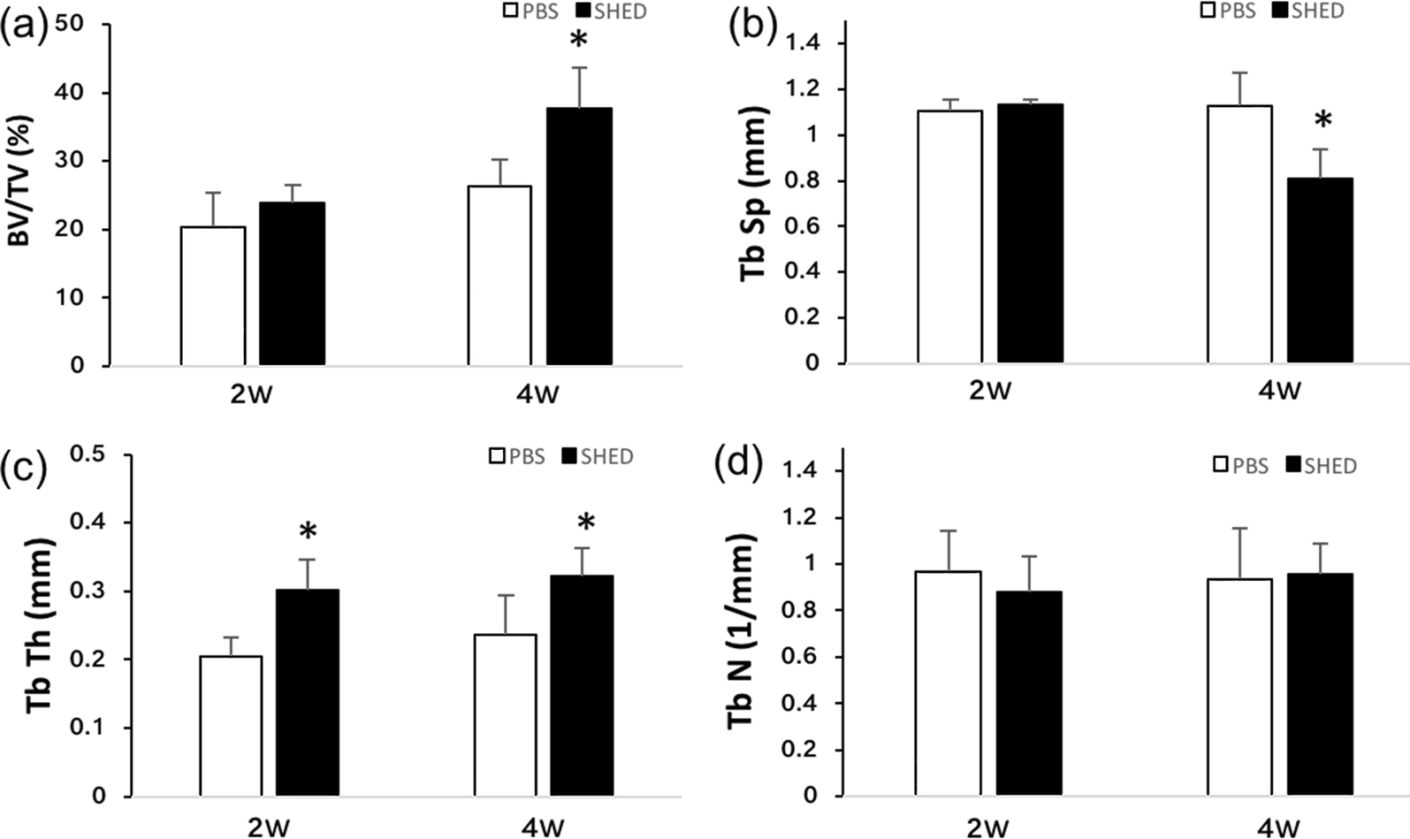
Analysis of the ratio of BV/TV, TbSp, TbTh and TbN in the region of interest. **a** BV/TV. **b** TbSp. **c** TbTh. **d** TbN. *BT/TV* bone volume fraction, *TbSp* trabecular separation/spacing, *TbTh* trabecular thickness, *TbN* trabecular number. *p < 0.05

Micro-CT scanning indicated that all the implants were surrounded by bone (Fig. 8). However, the 3D reconstruction of the peri-implant bone differed (Fig. 9). Compared with the control group, the peri-implant bone volume in the SHEDs group was higher at 4 weeks (37.67 ± 6.08 vs. 26.23 ± 4.04), and the bone trabecula was thicker and denser (Fig. 10 and Table 5), suggesting better osseointegration.

**Table 5:** Analysis of the ratio of BV/TV, TbSp, TbTh and TbN in the region of interest: 2-week healing, 4-week healing

Group	BV/TV 2 W (%)	BV/TV 4 W (%)	TbSp 2 W (%)	TbSp 4 W (%)	TbTh 2 W (%)	TbTh 4 W (%)	TbN 2 W (%)	TbN 4 W (%)
PBS	20.29 ± 5.06	26.23 ± 4.04	1.10 ± 0.05	1.13 ± 0.14	0.20 ± 0.03	0.24 ± 0.06	0.96 ± 0.18	0.94 ± 0.22
SHED	23.97 ± 2.49	37.67 ± 6.08	1.13 ± 0.03	0.81 ± 0.13	0.30 ± 0.04	0.32 ± 0.04	0.88 ± 0.15	0.96 ± 0.13

## Discussion

Osteoblasts play an important role in the osseointegration interface. In addition to endogenous cells, the addition of exogenous cells can further promote the osseointegration process [39], Wei Zhou et al. demonstrated that BMSCs sheets contribute to extensive bone and blood vessel formation in vivo after wrapping around implants, indicating that the implantation of BMSCs has good osseointegration potential [40]. Compared with BMSCs, dental-derived stem cells have received extensive attention in recent years due to their high proliferation and differentiation ability and less ethical issues [10, 30]. Francesca Diomede et al. have demonstrated that PDLSCs have osteogenic and angiogenic abilities on titanium surfaces [9]. Yoichi Yamada and Kenji Ito et al. found that the implantation of DPSCs can promote implant osseointegration [41, 42]. Compared with DPSCs and BMSCs, SHEDs produce much more osteoid and a rich network of collagen fibers [43], and exhibit higher bone regeneration ability, which may contribute to the better bone remodeling and osteoid deposition seen in the SHEDs group of this experiment.

The overall results of the current in vivo experimental study showed that the application of SHEDs promoted early osteogenesis around the implant. Compared with a control group, the SHEDs group showed better BIC%, more osteogenesis in the thread, better contact osteogenesis, and the formed trabecular bone structure was thicker and denser, and this advantage was more obvious at 4 weeks after implantation than at 2 weeks.

In this study, we found a significant difference in the amount of IB on the lingual side between the SHEDs and control groups, which may be due to differing microenvironments inside the thread. Because of the implantation of SHEDs and their high proliferation rate and osteogenic differentiation capability, the experimental group showed greater osteogenesis ability and more osteogenesis in the thread. In this experiment, a considerable quantity of SHEDs were pre-adhered to the implant thread. The implant thread comprised a chamber with numerous SHEDs and blood clots in it, since SHEDs can produce a large amount of bone in vivo [44], which could better promote the osteogenesis of IB and the increase in BIC%. Meanwhile, SHEDs have a superior proliferation rate, so the pre-adhered SHEDs in the implant could exponentially multiply in a short time, which can better promote osteogenesis. In addition, stem cells could be recruited to the injury site and contribute to the pool of osteoblasts that form peri-implant bone and participate in wound healing and osseointegration [45–48]. In this way, exogenous implantation of SHEDs could accelerate the efficiency of bone integration by directly increasing the number of functional cells in situ and by indirectly increasing the number of functional cells recruited.

There was no significant difference in the buccal IB between the experimental and control groups. This lack of statistical significance may be due to the limited sample size or the thinner buccal bone plate compared with that on the lingual side. Studies have shown that when the thickness of the buccal bone plate is less than 1.5 mm, bone resorption is more likely to occur [49]. The bone resorption of the buccal bone plate weakened the difference between the test and control groups.

After implant placement, there are two modes of bone formation, called distance osteogenesis and contact osteogenesis [50]. In distance osteogenesis, bone is initially formed on the surface of the mature bone tissue around the implant socket and grows toward the surface of the implant. In contrast, the growth pattern in contact osteogenesis proceeds from the surface of the implant to the surface of the bone tissue around the implant socket. In this case, the implant surface will be in direct contact with the newly formed woven bone. Compared with the distance osteogenesis model, contact osteogenesis leads to faster biological anchoring of the implant [51] and thus provides greater mechanical stability of the implant at an early stage than distance osteogenesis does. Rapidly establishing the necessary conditions for contact osteogenesis accelerates bone formation. Abrahamsson et al. [51] observed new bone formation by the first weekend after implant placement. The rate and extent of bone healing depend on the degree of contact osteogenesis on the surface of the implant [2]. A higher BIC% may indicate better contact osteogenesis. The higher percentages in Total BIC% at 2 and 4 weeks in the SHEDs group compared with that in the control group may be due to the osteoinductive properties and high proliferation of SHEDs on the implant surface, which is consistent with studies reporting that SHEDs can generate large amounts of bone in vivo [44]. Since soft tissue formation at the BIC interface has a negative impact on fixation [43], early bone covering of the implant surface is thought to play an important role in preventing soft tissue penetration [52]. Compared with the control group, the better IB and BIC% found in the SHEDs group indicates better contact osteogenesis, which affects the quality and rate of bone healing and promotes osseointegration.

There were limitations in this study. In the histological analysis, only the buccal-lingual section was studied, so any structural changes that occurred in the mesio-distal dimension were missed. However, this limitation has been partially compensated by micro-CT analysis, which evaluates 360 degrees around the implant surface to better indicate the bone regeneration [53, 54]. It should also be noted that despite the limits of the study, the SHEDs group had significantly more bone around the implant at 4 weeks, with a denser and thicker trabecular bone structure, a decreased trabecular bone separation rate, and better osteogenesis than that found in the control group.

## Conclusions

In summary, SHEDs-loading the implant prior to implantation improved the early osseointegration around the implant in the beagle dogs, increased bone formation around the implant and in the thread, and resulted in thicker and denser trabecular bone. These results suggest that SHEDs can promote early osteogenesis around implants and may provide a perspective for stem cell therapy in future clinical trials of implants.

## Data Availability

The datasets used and/or analysed during the current study are available from the corresponding author on reasonable request.

## Abbreviations

SHEDs: Stem cells from human exfoliated deciduous teeth
BIC: Bone in contact
MSCs: Mesenchymal stem cells
BMMSCs: Bone marrow mesenchymal stem cells
DPSCs: Dental pulp stem cells
PBS: Phosphate-buffered saline
Micro-CT: Micro-computed tomography
VOI: Volume of interest
BT/TV: Bone volume fraction
TbTh: Trabecular thickness
TbN: Trabecular number
TbSp: Trabecular separation/spacing
BF: Peri-implant bone fraction
IB: Interthread bone
